# Prediction of Bioactive Compounds and Antioxidant Activity in Bananas during Ripening Using Non-Destructive Parameters as Input Data

**DOI:** 10.3390/foods13142284

**Published:** 2024-07-20

**Authors:** Angela Vacaro de Souza, Vitória Ferreira da Silva Favaro, Jéssica Marques de Mello, Vinicius Canato, Diogo de Lucca Sartori, Fernando Ferrari Putti, Yasmin Saegusa Tadayozzi, Douglas D’Alessandro Salgado

**Affiliations:** School of Science and Engineering, São Paulo State University (UNESP), Campus Tupã, Tupã 17602-496, SP, Brazil; vitoria.favaro@unesp.br (V.F.d.S.F.); jessica.mello@unesp.br (J.M.d.M.); vinicius.canato@unesp.br (V.C.); diogo.sartori@unesp.br (D.d.L.S.); fernando.putti@unesp.br (F.F.P.); yasmin.tadayozzi@unesp.br (Y.S.T.); douglas.salgado@unesp.br (D.D.S.)

**Keywords:** *Musa* sp., predictive model, linear regression, non-destructive food analyses

## Abstract

Vegetable quality parameters are established according to standards primarily based on visual characteristics. Although knowledge of biochemical changes in the secondary metabolism of plants throughout development is essential to guide decision-making about consumption, harvesting and processing, these determinations involve the use of reagents, specific equipment and sophisticated techniques, making them slow and costly. However, when non-destructive methods are employed to predict such determinations, a greater number of samples can be tested with adequate precision. Therefore, the aim of this work was to establish an association capable of modeling between non-destructive—physical and colorimetric aspects (predictive variables)—and destructive determinations—bioactive compounds and antioxidant activity (variables to be predicted), quantified spectrophotometrically and by HPLC in ‘Nanicão’ bananas during ripening. It was verified that to predict some parameters such as flavonoids, a regression equation using predictive parameters indicated the importance of R^2^, which varied from 83.43 to 98.25%, showing that some non-destructive parameters can be highly efficient as predictors.

## 1. Introduction

Subjectivity in vegetable classification processes arises from the use of visual parameters, whose patterns are defined and tabulated in tables and figures. When put into evidence, this subjectivity can lead to problems between producers/sellers and inspection bodies since pricing is linked to this standardization. Furthermore, there are elements of oral arguments that can be taken into account during inspection, adding more quality and/or less objectivity to the processes. In this context, the classification of the fruit ripening stage is of paramount importance, as it prevents the products from presenting serious defects, such premature ripening, advanced ripening, or the presence of mild and serious defects that may reach the consumer’s table. These peculiarities are inherent to each culture, variety and ripening stage of the fruit, making it difficult, if not impossible, to select a general technique that applies to all cases [1].

For being a climacteric fruit, bananas undergo changes throughout the ripening process that include the following: changes in color, going from green to yellow with brown spots during the senescence period; the degradation of starch and synthesis of sugars, causing profound changes in their flavor and aroma; the degradation of pectic substances and loss of water, making them softer; and changes in the compounds related to secondary metabolism, e.g., the synthesis of bioactive compounds including flavonoids. A study by [2] showed that bananas contain different amounts of flavonoids during ripening, and the flavonoids quantified showed an increase in prophylaxis during this phase, with quercetin and genistein standing out. The quantification of bioactive components present in vegetables can be extremely elaborate, requiring biochemical extraction procedures in fresh materials, in addition to specific standard reagents and products, subsequent HPLC and/or spectrophotometric measurements, and specialized personnel. Such sophistication generally implies high costs for carrying out analyses, especially when greater precision is required, due to the number of samples or repetitions.

Only in recent years has the introduction of imaging, e.g., near-infrared, fluorescence, mid-infrared and multispectral/hyperspectral imaging [3,4], reflectance and colorimetry (stored in RGB, XYZ, CMYK, HEX, and CIELAB formats) [5] led to the increasing adoption of non-destructive devices to estimate food quality, making spectrophotometric methods based on the non-destruction of plants highly recommended. Such methods provide an increased number of samples and repetitions, as well as savings in raw materials, reagents and time optimization, with color measurement devices being used in these cases to determine color attributes in the CIELAB space [6]. Imaging systems are designed to further align with human color perception, considering the non-linear nature of our visual system. These color spaces attempt to create a consistent and uniform color model that mimics human color perception [7]. Thus, color is an important classification component attributed to various quality factors [8].

To accurately predict the pattern of changes in all quality parameters for a given plant variety can be very challenging. By transforming a subjective and slow method into an objective and agile one, errors can be reduced, causing the producer to be remunerated more assertively according to the quality of their products and helping the consumer to consume more suitable products. Given this context, this work aimed to establish an association capable of modeling between non-destructive—physical and colorimetric aspects (used herein as predictive variables)—and destructive determinations—bioactive compounds and antioxidant activity (used herein as variables to be predicted), quantified spectrophotometrically and by HPLC in ‘Nanicão’ bananas during ripening.

## 2. Materials and Methods

### 2.1. Vegetal Material

‘Nanicão’ bananas, triploid of *Musa acuminata* (AAA) from the Cavendish subgroup, were harvested from a commercial producer in the municipality of Tupã, state of São Paulo, Brazil, whose climate is Cwa (i.e., dry-winter humid subtropical climate), according to the Köeppen classification. Based on the observation of similarities found between the fruits, they were harvested and separated into four different groups according to the stages of ripeness: stage 2 (green with light yellow traces); stage 4 (more yellow than green); stage 6 (yellow); and stage 7 (yellow with brown spots) according to the scale proposed by [9]. The non-destructive (predictive) and destructive (predicted) determinations were performed in the Quemistry Laboratory belonging to the belonging to the School of Science and Engineering, Tupã, state of São Paulo, Brazil, on 30 bananas of each ripening stage, totaling 120 samples.

### 2.2. Non-Destructive Determinations—Predictive Variables

Non-destructive physical and colorimetric determinations (herein used as predictive variables) were performed on intact bananas.

#### 2.2.1. Physical Analysis

We recorded weight, measured using a scale (g); length and average diameter (cm); and texture (firmness), measured at three points, i.e., in the equatorial, apical and basal regions, by means of a texture analyzer/digital penetrometer (VICTOR-GY4) at a penetration distance of 10 mm using a straight circular tip with a diameter of 3.50 mm (N).

#### 2.2.2. Peel Colorimetry by Digital Imaging

We performed this step at three points (i.e., equatorial region and at the two ends 3 ± 1 cm away from the median region) in each fruit based on the analysis of pixel samples taken from digital images to obtain the parameters of the CIE L*a*b* color system, which consists of luminosity values (L*), a chroma measurement on the red-green axis (a*) and a chroma measurement on the yellow-blue axis (b*), using a Canon digital single-lens reflex (DSLR) camera (model EOS 90D) with a 24 mm fixed lens (Canon, model EF-S 24 mm f2.8 STM). This set was supported and positioned with the aid of a tripod on the surface of a bench with black fabric to standardize the image background at an angle of 90°.

**Figure 1:**
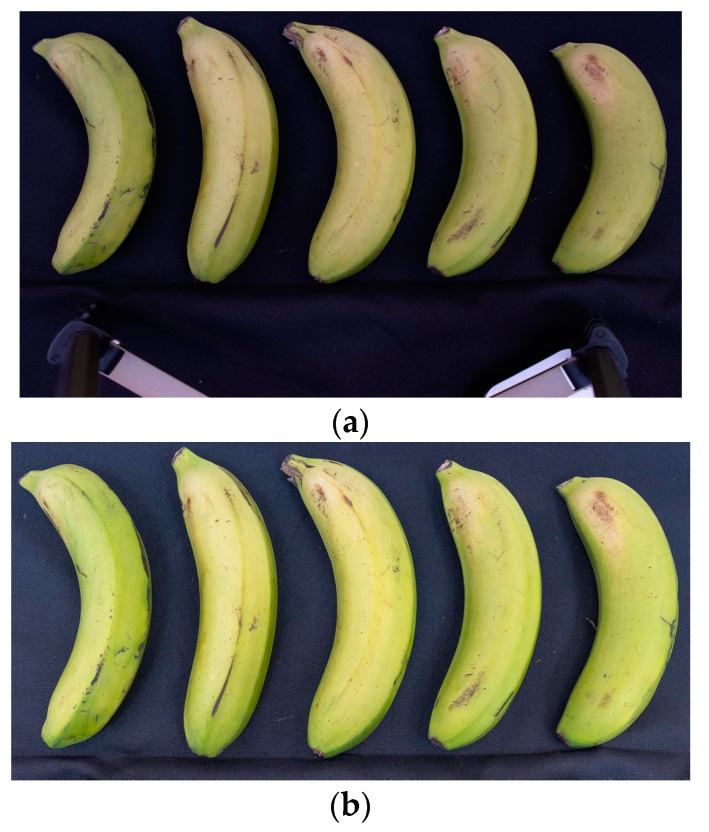
Image of the RAW file captured directly by the camera, demonstrating how the images were obtained on the bench (**a**); and image file after digital development and framing adjustment (**b**).

The bananas were arranged in up to five specimens at a time to compose each image, with each fruit being previously identified with a number and arranged in order (Figure 1). To capture the images, a light source from a LED lamp with a color temperature of 6500 K was used. The camera was set to manual exposure mode at an ISO sensitivity of 500, diaphragm aperture of f/5.6 and exposure time of 1/80 s, and it was adjusted to a RAW image quality (original files in CR3 format) at a resolution of 6960 × 4640 pixels (Figure 1a). The RAW files underwent a digital development process using Adobe Photoshop Lightroom Classic software version 10.1.1, where standard and framing adjustments were made to all files, followed by cropping of the original image to a size of 6960 × 3504 pixels. Afterwards, the images were exported to a PSD file using Adobe RGB color space (1998) at a depth of 16 bits/component (Figure 1b).

**Figure 2:**
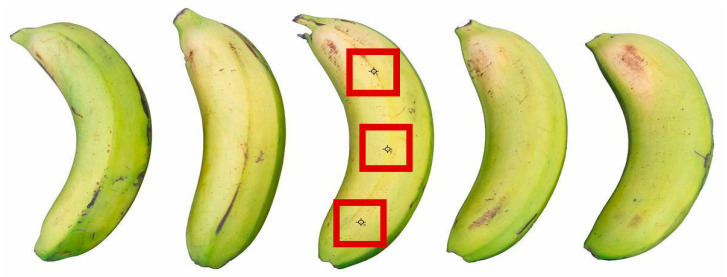
Final image and indication of the points to obtain L*, a* and b* values on the fruit peel surface.

To obtain the values of L*, a* and b* in the images of the fruit peel surface, Adobe Photoshop version 22.2.0 was used. To this end, the image mode of all previously exported PSD files was converted from RGB to LAB colors at 16 bits/channel. For color sampling, the Color Classifier tool was employed, defining the sample size as an average of 101 by 101 pixels (Figure 2).

**Figure 3:**
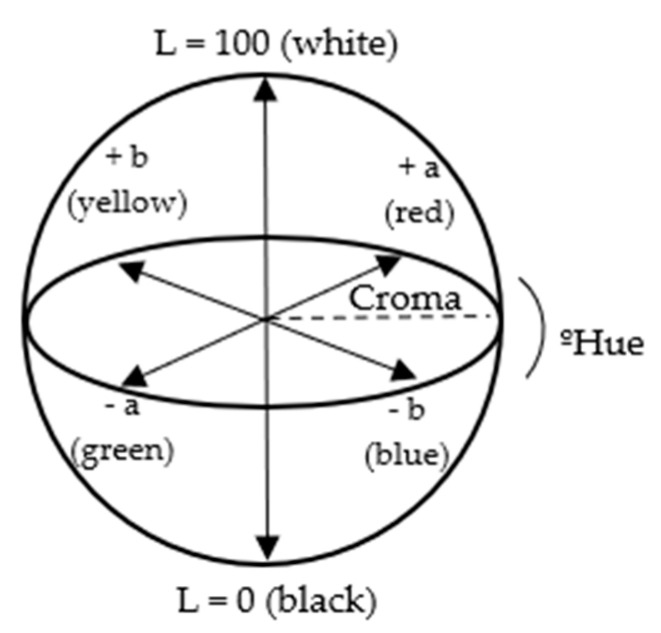
Diagram of the CIELAB color space, where L* indicates luminosity, a* and b* are chromaticity coordinates, chroma (C*) represents color saturation and the hue angle represents hue.

The color data were stored in the CIE L*a*b* format, where L* represents the relative degree of brightness, the chromatic descriptors +a* and −a* indicate the degree of red and green colors, respectively, the chromatic descriptors +b* and −b* demonstrate the degree of yellow and blue colors, C* represents the chromaticity [10] and h° is the hue angle (Figure 3). Based on these data, the degreening [11], yellowness [12] and color indexes [13] were also calculated, according to the following formulas:

C* = ((a*)^2^ + (b*)2)^1/2^ (1)

h° when a* is positive = 90(tan^−1^(b*/a*)) (2)

h° when a* is negative = tan^−1^(b*/a*) × (−1) + 90 (3)

Chromaticity: (a*2 + b*2)^1⁄2^ (4)

Degreening index: (1000 × a)/(L × b)(5)

Yellowness index: 142.86b*/L*(6)

Color index: CI = 1000 × a*/L × b*(7)

### 2.3. Destructive Determinations—Predicted Variables

Destructive determinations were carried out on the fruit pulp and used as predicted variables. The original data regarding the quantifications carried out are published [2].

Quantification of bioactive compounds and antioxidant activity: The spectrophotometric determinations were phenolic compounds, quantified according to [14]; chlorophyll a and b, anthocyanins and carotenoids, according to the methodology adapted from [15]; total antioxidant activity by DPPH, according to [16] with some modifications; antioxidant activity by FRAP (ferric reducing antioxidant power), measured according to [17]; and total sugar [18], realized in triplicate.

Quantification of flavonoids and ascorbic acid: The analyses were carried out on a Shimadzu Prominence high-performance liquid chromatography (HPLC) system equipped with an SPD-20 ultraviolet detector programmed to operate at wavelengths of 240 nm and 350 nm. The curve points were obtained by injecting 5 µL of the standard solution at different concentrations using an automatic sampler model SIL-10AF, and the system was equipped with a Zorbax Eclipse Plus C18 2.1 × 100 mm, 3.5 um column (AGILENT) at 30 °C. The mobile phase was composed of a 0.1% formic acid solution (A) as the polar solvent and acetonitrile (B) as the apolar solvent. The chromatographic method took place over a period of 35 min in gradient mode following this schedule: 0 min—20% B; 25 min—80% B; 25.01 min—20% B; 35 min—20% B.

The calibration curve was constructed by diluting a 10 mg mL^−1^ stock solution containing all the standards, obtaining the following concentrations: 100 µg mL^−1^, 200 µg mL^−1^, 300 µg mL^−1^, 400 µg mL^−1^, 500 µg mL^−1^, 600 µg mL^−1^, 700 µg mL^−1^, 800 µg mL^−1^ and 900 µg mL^−1^. All the solutions were prepared independently and analyzed on the HPLC system in triplicate.

For the samples, 1 g of banana was freeze-dried in a LIOBRAS K105 freeze-dryer, basically composed of a control panel, drying chamber, heating tray, steam condensation chamber, compressor, vacuum system, heat exchanger, air dryer and drain outlet. The final pressure applied was around 200 mmHg and the temperature of the drying chamber was −103 °C. After freeze-drying, the flavonoids and ascorbic acid were extracted with 5 mL of 50% methanol/water solution. The samples were centrifuged and filtered through a 0.45 µm nylon syringe filter before being injected into the HPLC.

The standard solutions of ascorbic acid, rutin, daidzein, quercetin and genistein were prepared using reagents of analytical purity (St. Louis, MO, USA, Sigma-Aldrich), P. A. and ultrapure water. HPLC grade acetonitrile (Muskegon, MI, USA, Honeywell) and formic acid (Diadema, SP, Brazil, Synth) were used to prepare the mobile phase.

To present the results obtained, the concentration of the liquid extract (from the banana) from the HPLC in μg mL^−1^ was multiplied by the volume of extracting solution (5 mL), and the total mass of the substances analyzed was found. This value was divided by the mass of fresh banana (1 g), before being freeze-dried, and the final results were expressed in mg g^−1^ of fresh matter. The chromatogram and raw data for these analyses can be found in [2].

The method used was validated according to the following criteria [19]: selectivity, linearity, sensitivity, detection limit (DL), quantification limit (QL) and recovery. Table 1 shows the method validation parameters.

Linearity, a measure of the relationship between the concentration of an analyte and its analytical response, is given by the angular coefficient of the calibration curve (r); the closer it is to 1 expresses the linear relationship between the series analyzed. The r values obtained in Table 1 show that all the analytes have a linear relationship.

The selectivity of the method was measured by adding a standard. An aliquot of the stock solution was added to the sample matrix, and we checked for changes in the retention time of the chromatogram. Figure 1 shows a comparison of the retention times. It can be seen that there was no significant change, indicating that the method is selective.

The limit of detection (LD) shows the minimum concentration at which the analyte can be detected. The values express the signal-to-noise ratio and are acceptable when they produce an analytical signal three times greater than the noise of the equipment. The LD values for the method in question ranged from 4.35 µg mL^−1^ to 1.53 µg mL^−1^, the highest being for rutin and the lowest for genistein, and the detection limit for ascorbic acid was 2.89 µg mL^−1^, for daidzein 1.56 µg mL^−1^ and for quercetin 1.84 µg mL^−1^. The limit of quantification (LQ) is the lowest concentration at which the analyte can be quantified, with the values varying from 4.64 µg mL^−1^ for genistein to 13.18 µg mL^−1^ for rutin. The LQ values were accepted when they expressed approximately three times the LD value. The LD obtained for ascorbic acid was 8.77 µg mL^−1^, for daidzein 4.72 µg mL^−1^ and for quercetin 5.57 µg mL^−1^.

Recovery was assessed by fortifying the sample with a standard at a known concentration. Recovery was calculated to check the agreement between the theoretical value of a concentration and the measurement obtained by the method. The recovery values in Table 1 range from 98.6% to 104.4%, and for all analytes, the recovery was within the acceptable limit.

### 2.4. Data Analysis

The variables were first discriminated between predictive and predicted, thus facilitating the selection of the best associations that could lead to more promising models. To this end, the statistical data analysis was divided into four parts: (a) fitted regression curves, which describe the behavior of quality parameters as a function of ripening stages, as well as their respective coefficients of determination (R^2^), also called coefficient of determination; (b) pre-selection through the analysis of main components; (c) selection through two-by-two crossings and by calculating the Pearson’s correlation coefficient; and (d) modeling through linear regression (linearization).

#### 2.4.1. Linear Regression Analysis—Parameters versus Ripening Stages

Individual models were created by fitting a line to the observed data. The regression models described the connection between the variables, in this case the fruit ripening stages, X (independent variable), and each dependent variable, Y (non-destructive (predictive) and destructive (predicted) parameters). The R^2^ values obtained represent the average change in the variable response for one unit of change in the predictive variable, keeping the other predictors constant in the model.

#### 2.4.2. Principal Component Analysis (PCA)

As an initial analysis and in order to establish a first relationship between parameters, the predictive and predicted variables were discriminated by subjecting them to a multivariate analysis called principal component analysis. Through this method, it was possible to pre-select the predictive and predicted variables most likely to have strong associations, consequently resulting in promising models.

#### 2.4.3. Correlation Analysis

The pre-selection performed through PCA allowed for the creation of a table to find correlations between predictive and predicted variables. As a consequence, it was possible to make a finer selection through Pearson’s correlation, which quantifies the linear association between two variables. The most refined selection criterion was established by verifying correlations with module values greater than 0.7 and adopting a significance level of 5% (α = 5%). This work adopted the concept of correlation developed by [20], where values between 0.50 and 1 can be interpreted as strong.

#### 2.4.4. Linear Regression Analysis—Non-Destructive versus Destructive Parameters

Lastly, the best fitting regression equations were created using the predictive variables as input data and the predicted variables as output data. In order to find the best equations, several tests were carried out with more than one predictive variable, preferably those that did not require calculations to be determined. Minitab software version 18.1 (Minitab Inc., Munich, Germany) was used for both analyses.

## 3. Results and Discussion

As a first approach, the most appropriate models were created for each parameter evaluated individually in order to determine their respective performances in the different ripening stages of bananas. The models were registered in terms of a straight-line equation and a regression coefficient (R^2^) (Table 2). As noted, throughout the ripening stages, there was a reduction in all physical parameters, i.e., pigments, phenolic compounds, antioxidant activity by DPPH and quercetin. The performance relative to these same determinations was verified by [2,21], who studied ‘Nanicão’ bananas at different ripening stages and showed a drop in antioxidant activity and phenolic compounds after stage 4. As it is a climacteric fruit, it is known that such alterations make its post-harvest life relatively short because of the marked physical and chemical changes that can occur, such as the softening of tissues caused by the degradation of cell wall components, the loss of water and reserves due to the accentuated respiratory process, the degradation of chlorophyll and the synthesis of other pigments with changes in the peel color and subsequent degradation during senescence in overripe fruits [22].

In their work, ref. [23] noticed a 5% reduction in the diameter and around 8% in the weight of bananas during ripening, while other quality parameters such as ascorbic acid and some flavonoids showed an increase. A similar pattern was reported by [24] when studying *Musa nana* Lour vs. Dwarf Cavendish bananas. According to the authors, there was an increase of almost 90% in ascorbic acid content during the fruit ripening stages.

**Figure 4:**
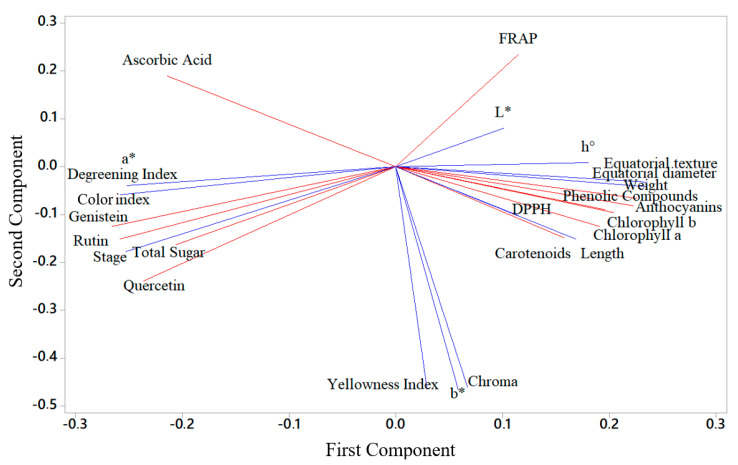
Two-dimensional projection of non-destructive (predictive) and destructive (predicted) parameters analyzed in fruits at stage 2, stage 4, stage 6 and stage 7, respectively. Non-destructive (predictive—in blue) and destructive (predicted—in red).

A PCA model combining non-destructive (predictive—in blue) and destructive (predicted—in red) determinations was created, resulting in a data matrix of 120 observations (samples) and 24 measured variables—12 predictive and 12 predicted. As illustrated in Figure 4, there are two groupings of data close to the horizontal axis (first component), which indicates that the determinations (both predictive and predicted) tend to have a strong positive or negative correlation. The second component was best represented by parameters related to fruit color. This evaluation is interesting for this type of verification as it is an unsupervised method to extract a realistic result of the association between the banana ripening phases and the changes the fruit experiences. The figure also reveals the proportion of how much the two components (axes) explain the relationship between the variables on a scale from 0 to 1 (i.e., 0.640). In relation to the variables that show a reduction in their respective levels as the ripening stage advances, there is an approximation of these values on the left side of the figure, e.g., flavonoids. An opposite behavior, that is, a reduction in levels demonstrated by parameters such as length, weight, diameter, pigments and total sugars, is observed in the grouping on the right side of the Figure 4.

Plant maturation and ripening are coordinated by hormones and signals and genetically programmed to allow for the transformation of the immature organ to the mature stage, where the degradation and synthesis of metabolites are inherent to the process, as well as the strong associations and correlations between parameters. A number of biochemical changes occur in the banana during this process, including the conversion of starch into sugars, changes in the color of the peel and pulp with the synthesis of other pigments and degradation of chlorophylls, changes in the cell wall causing softening, and variations in the concentration of volatiles and organic acids in order to increase attractiveness. Table 2 shows a strong relationship between physical and colorimetric parameters and the quantified flavonoids rutin, quercetin and genistein. Since the quantification of flavonoids requires standards, reagents, sophisticated equipment and trained personnel to be precise, the association between them and non-destructive quality parameters makes the results extremely valuable from an economic and environmental point of view, as there is a reduction in the use of reagents, in addition to the possibility of quantification on a classification line or in the field, which is decisive for the harvest.

From this analysis, the correlations between predictive and predicted determinations were determined individually in order to verify whether there is an association, that is, whether it is possible to predict a destructive determination by having access only to a non-destructive one. The results can be found in Table 2. As previously mentioned, in this work, the concept of correlation used was that developed by [20], where values between 0.50 and 1 can be interpreted as strong—demonstrated in the tables with colors ranging from light (above 0.50) to dark gray (above 0.70), according to the intensity of correlation. Values close to 1 (regardless of the sign) represent a greater degree of linear statistical dependence between the variables, while values close to zero indicate a weak relationship (Table 3).

When studying ‘Cardaba’ bananas, ref. [25] found a positive association (R^2^ ≥ 0.93) between the ripening stage and physical, optical and quality parameters, soluble solids, mass loss, pulp proportion, acidity, and color indices L*, a*, C, b* and ΔE. On the other hand, ref. [26] observed that soluble solids correlated positively with titratable acidity (R^2^ ≥ 0.808) and soluble solids/titratable acidity (‘Ratio’) (R^2^ = 0.932), and negatively with pH (R^2^ = −0.881). The authors emphasized the importance of the fruit peel color through the verification of color parameters to explain other quality attributes during storage since the color parameters L*, a* and b* showed a strong correlation with most quality characteristics of ‘*Musa acuminata* L. AAB group vs. Fard’ bananas, with these colorimetric parameters being potential predictors of quality attributes in this case.

Ref. [27] reported significant results when studying ‘Cavendish’ bananas and their color changes during shelf life, with the aim of evaluating the images collected as a quick and non-destructive alternative for effectively predicting quality indices. After different tests, the artificial neural network was used to estimate fruit firmness from the images, with a coefficient of determination between the experimental and predicted values of 95.01%. The corresponding value was 99.50% for the support vector regression (SVR) machine learning method, with the best SVR result being the prediction of banana pH (R^2^ = 99.74%). In this context, advances in non-destructive image-based approaches and other simple quality determination techniques can mitigate the dependence on human and subjective visual identification and laboratory analyses to classify the ripening stage of fruits. Some of these systems employ classification algorithms that use different color features or a combination of them with other ripening indicators, such as those used herein for effective performance [28].

**Figure 5:**
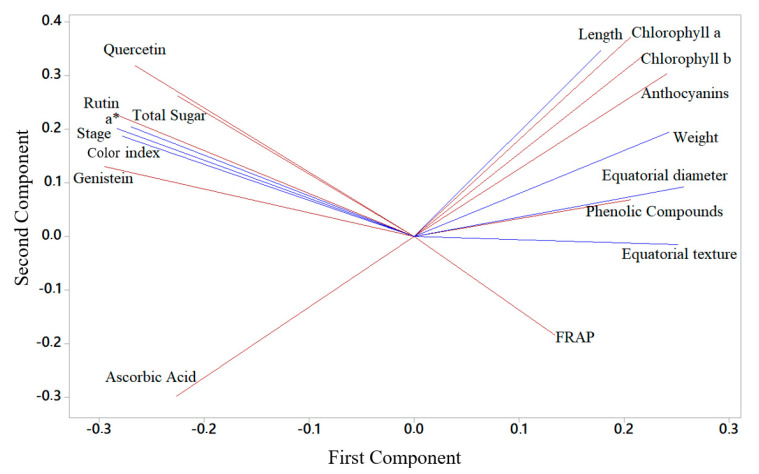
Two-dimensional projection of non-destructive (predictive) and destructive (predicted) parameters selected as most in fruits at stage 2, stage 4, stage 6 and stage 7, respectively. Non-destructive (predictive—in blue) and destructive (predicted—in red).

From the table, it was possible to verify which parameters (non-destructive and destructive) had strong associations with each other. Then, a new PCA was carried out in order to verify how much both components could explain the relationship between the variables on a scale of 0 to 1—in this second case, the value was 0.892 (higher than that previously found). The positions of the vectors in Figure 5 show that the evaluated attributes were redivided into two larger groups and grouped close to the horizontal axis.

From these associations, regression equations were created with the aim of determining the selected predicted determinations (i.e., total sugar, rutin, quercetin and genistein) based on the predictive ones (Table 3). For the other determinations, the regression equations developed resulted in low R^2^ values, evidencing that in these cases, the associations between the predictive and predicted variables were weak.

Ref. [29] observed color and texture changes in Musa AAA (variety ‘Maritu’) during the ripening stage and developed mathematical models using time- and temperature-dependent variables. Similarly, texture and visual changes in ‘Grand Naine’ bananas at different ripening stages were modeled using texture and visual modifications and sensory data. The results obtained pointed to stage 6 as a critical point of quality decline, while the instrumental analysis revealed a trend that extended to stage 7 [30], showing that there may be divergences between the results depending on the evaluation method used. While there are unequivocal similarities between the climacteric of different banana varieties, studies aiming to use existing relationships and associations between destructive and non-destructive parameters are necessary since the work found in the literature is still incipient.

Regression models were created in an attempt to predict destructive parameters using non-destructive variables (Table 4). To this end, the results presented in Table 2 are used as a basis. For total sugar, the following parameters were selected: stage and equatorial texture by means of the stepwise method. The variables with the best adherence to the model were pre-selected, mainly the variable stage, which was selected as a highly significant predictor, and HUE, considered a less-important variable to explain total sugar.

However, the residue analysis indicated the need to transform the variable total sugar into its square root, which resulted in an increase of five percentage points (from 63.38% to 68.98%) in the coefficient of determination, a substantial reduction in the model standard deviation and the proposition of a leaner model, with just a single predictive variable (stage). Although regression equations were developed individually for each selected predictive parameter, the R^2^ values obtained were considered low (stage: 67.37%; and equatorial texture: 25.93%). For all destructive parameters, more appropriate regression equations were elaborated, according to Table 4.

For rutin, when evaluated individually, the non-destructive parameters exhibited the following R^2^ values: stage—83.43%, equatorial texture—68.68%, a*—81.83% and color index—82.45%. When an equation was created taking into account all the previously described parameters with high associations, the R^2^ increased to 95.60%.

Regarding quercetin, when evaluated individually, the non-destructive parameters stage, a* and color index showed the respective R^2^ values: 92.82%, 68.31% and 72.05%. Due to the simplicity of determination, Table 4 presents the regression equation for stage as a predictor of this flavonoid. When all destructive parameters were used with the stepwise model, the R^2^ increased to 95.60%.

Lastly, for genistein, when the regression model was applied individually based on non-destructive parameters (Table 4), it was found that the variable stage exhibited an R^2^ of 97.45, being considered the most appropriate predictive parameter for predicting this destructive parameter. The other R^2^ values found for weight, diameter, texture, a* and color index were 50.47%, 57.98%, 49.07%, 69.39% and 75.71%, respectively.

The parameter texture was removed since it showed lower variance inflation factors (VIF), resulting in an R^2^ of 98.25%. The VIF is a statistical measure that indicates the degree of multicollinearity present in a regression model. This factor was calculated for each independent variable and provided an estimate of the influence of multicollinearity on the precision of coefficient estimates, basically measuring how much the variance of a regression coefficient increased due to multicollinearity with the other independent variables, which in these cases were evaluated and removed from the equations according to their influence. In order to make the model leaner, a test was carried out only with stage and weight (parameters that are easy to detect when classifying vegetables), resulting in an R^2^ value of 97.96% (Table 4)—a value a little superior to that in the model when only stage was used.

After obtaining the regression equations, the performance of the predictive model was evaluated. As an example, it will be compared with the results obtained in a previous study by [2]. When quercetin was quantified in the laboratory, the values obtained were 0.0365 (stage 2), 0.0365 (stage 4), 0.0366 (stage 6) and 0.0366 (µg g^−1^) (stage 7). When using the equation, we obtained 0.364354 + 0.000225 for this stage, and the quantified values ranged from 0.0368 to 0.0380.

## 4. Conclusions

Color is an important attribute for determining plant classification and standardization, indicating quality and often determining its valuation. The regression models based on non-destructive predictive parameters used herein proved to efficiently infer the selected destructive predicted parameters, with R^2^ values ranging from 68.98 to 98.25% proving to be highly efficient for this purpose. It is worth noting that the simplicity, ease and efficiency of the model show a great potential for predictions to occur in the field with the aid of specific equipment for image capture or in the production and classification line.

The choice of the mathematical model is simple and is carried out in software with parameters that are very simple to use, and the visual evaluation of the fruit (stage) in relation to the stages of ripeness would already be sufficient in estimating these complex parameters.

The predictive parameters reported in this work are easy to determine and do not require the use of sophisticated equipment, reagents, standards and qualified technicians, making them even more interesting for further investigation.

## Figures and Tables

**Table 1:** Validation parameters.

Analyte	Retention Time (min)	Intersection	Slope	r	LD (μg mL^−1^)	LQ (μg mL^−1^)	Recovery (%)
Ascorbic acid	0.97	−5.00 × 10^4^	5038.14	0.9972	2.89	8.77	103.2
Rutin	15.646	1.89 × 10^5^	4102.21	0.9944	4.35	13.18	104.4
Daidzein	18.668	5.41 × 10^5^	9365.79	0.9965	1.56	4.72	103
Quercetin	19.973	6.24 × 10^5^	9292.45	0.9977	1.84	5.57	100.9
Genistein	21.011	3.35 × 10^5^	8823.13	0.9997	1.53	4.64	98.6

**Table 2:** Most suitable model to describe the performance of non-destructive (predictive) and destructive (predicted) parameters evaluated in fruits at stage 2, stage 4, stage 6 and stage 7, respectively.

Parameter	Most Suitable Model	R^2^
Weight (g)	y = −4.6336x^2^ + 27.097x + 158.07	0.8856
Lenght (cm)	y = −0.2152x^2^ + 1.575x + 13.943	0.7306
Equatorial diameter (cm)	y = −0.0316x^2^ + 0.1671x + 4.1789	0.9662
Equatorial texture (N)	y = −3.278x^2^ + 24.837x − 3.1559	0.8547
Chlorophyll a (mg 100g^−1^)	y = −0.0008x^2^ + 0.0059x + 0.004	0.8852
Chlorophyll b (mg 100g^−1^)	y = −0.001x^2^ + 0.0065x + 0.0092	0.891
Anthocyanins (mg 100g^−1^)	y = −0.0082x^2^ + 0.0582x + 0.0591	0.9631
Carotenoids (mg 100g^−1^)	y = −0.0017x^2^ + 0.0137x + 0.0204	0.8851
Phenolic compounds (mg of gallic acid 100 g^−1^)	y = −8.8358x^2^ + 68.832x − 31.633	0.9361
DPPH (%)	y = −2.1792x^2^ + 16.733x + 30.145	0.99
FRAP (mol g^−1^ FeSO_4_)	y = 0.0029x^2^ − 0.0316x + 0.0945	0.9991
Total sugar (g 100g^−1^)	y = −0.056x^2^ + 2.9597x − 2.2725	0.9637
Rutin (mg g^−1^)	y = 0.0568x − 0.1503	0.8343
Quercetin (mg g^−1^)	y = −0.0001x^2^ + 0.001x + 0.3639	0.9251
Genistein (mg g^−1^)	y = 5 × 10^−5^x^2^ – 2 × 10^−5^x + 0.2339	0.9989
Ascorbic acid (mg g^−1^)	y = 0.0866x^2^ − 0.6991x + 3.1541	0.8954
L*	y = −1.1417x^2^ + 4.645x + 80.158	0.8619
a*	y = 0.7083x^2^ + 1.6817x − 11.492	0.7952
b*	y = −1.4366x^2^ + 13.186x + 23.051	0.8782
°Hue	y = −1.1236x^2^ + 6.9196x + 87.928	0.898
Chroma	y = −1.4674x^2^ + 13.345x + 23.667	0.908
Color index	y = 0.1815x^2^ − 0.8191x − 1.3005	0.9396
Degreening index	y = 0.0002x^2^ − 0.0008x − 0.0013	0.9396
Yellowness index	y = −2.0028x^2^ + 18.959x + 45.136	0.8235

**Table 3:** Pearson’s correlation at a 5% confidence interval of non-destructive (predictive) and destructive (predicted) parameters evaluated in fruits at stage 2, stage 4, stage 6 and stage 7, where the correlation values greater than 0.500 or less than −0.500 are in light gray and values greater than 0.700 or less than −0.700 are in dark gray.

Parameters	Total Sugar (g 100 g^−1^)	Chlorophyll b (mg 100 g^−1^)	Anthocyanins (mg 100 g^−1^)	Phenolic Compounds (mg of Gallic Acid 100 g^−1^)	FRAP (mol g^−1^ FeSO_4_)	Rutin (mg g^−1^)	Quercetin (mg g^−1^)
Stages	0.772 0.000	-	-	-	−0.516 0.000	0.913 0.000	0.963 0.000
Weight (g)	-	0.557 0.000	-	-	-	−0.583 0.000	−0.548 0.000
Length (cm)	-	-	-	-	-	-	-
Equatorial diameter (cm)	-	0.548 0.000	0.571 0.000	-	-	−0.671 0.000	−0.637 0.000
Texture (N)	−0.829 0.000	-	0.589 0.000	0.663 0.000	-	−0.829 0.000	−0.636 0.000
a*	0.654 0.000	-	-	-	-	0.905 0.000	0.825 0.000
Color index	0.644 0.000	-	-	-	-	0.908 0.000	0.849 0.000

**Table 4:** Regression equations of non-destructive (predictive) and destructive (predicted) parameters evaluated in in fruits at stage 2, stage 4, stage 6 and stage 7.

Parameters	Regression Equation	R^2^
Total sugar	(0.1131 + 0.4040 Stage)^2^	68.98%
Rutin	−0.01503 + 0.05676 Stage	83.43%
Quercetin	0.0364354 + 0.000225 Stage	92.82%
Genistein	0.0233122 + 0.000432 Stage	97.45%
Genistein	0.0233716 + 0.000374 Stage − 0.000002 Weight + 0.000011 a*	98.25%
Genistein	0.0233576 + 0.000404 Stage − 0.000002 Weight	97.96%

## Data Availability

The original contributions presented in the study are included in the article, further inquiries can be directed to the corresponding author.
